# Supplementary material for: First report of blaOXA-181-carrying IncX3 plasmids in multidrug-resistant Enterobacter hormaechei and Serratia nevei recovered from canine and feline opportunistic infections
Source: Microbiol Spectr. 2024 Feb 6;12(3):e03589-23. doi: 10.1128/spectrum.03589-23 (PMC10913469; doi:10.1128/spectrum.03589-23)
Supplement: Supplemental material — Tables S1 to S3; Fig. S1 and S2. [file spectrum.03589-23-s0001.pdf]

**SUPPLEMENTAL MATERIAL**

**Table S1** Origin, clinical manifestation, and genome and plasmid characteristics of the six OXA-181-producing *Enterobacter hormaechei* and two OXA-181-producing *Serratia nevei* isolated from dogs and cats

| Strain | Species and subspecies | Host | Clinical Manifestation | Sequence type | Chromosome size | Intrinsic antimicrobial resistance genes on chromosome | Acquired antimicrobial mechanism(s) on chromosome | Number of plasmids | Plasmids (Incompatibility complex; size): Antimicrobial resistance genes |
|---|---|---|---|---|---|---|---|---|---|
| CUVET 18-121 | *Enterobacter hormaechei* subsp. *xiangfangensis* | Dog | Renal calculi | 182 | 4,731,337 | $bla_{\text{ACT-16}}$, *fosA* | *gyrA* (S83I) | 5 | **pCUVET18-121.1** (IncFIB: 165,063 bp): $bla_{\text{TEM-1}}$, $bla_{\text{OXA-1}}$, *aph(3'')-Ib*, *aph(6)-Id*, *aac(6')-Ib-cr5*, *qnrA1*, *arr-3*, *tet*(D), *catA2*, *catB3*, *sul1*, *sul2*, *dfrA14*<br>**pCUVET18-121.2** (IncR: 82,723 bp): $bla_{\text{OXA-2}}$, *aph(3'')-Ib*, *ant(2'')-Ia*, *cmlA10*<br>**pCUVET18-121.3** (IncX3: 51,479 bp): $bla_{\text{OXA-181}}$, *qnrS1*<br>**pCUVET18-121.4** (ColRNAI/Col440II: 5,411 bp): –<br>**pCUVET18-121.5** (ColRNAI: 2,495 bp): – |
| CUVET 19-891 | *Enterobacter hormaechei* subsp. *xiangfangensis* | Dog | Cystitis | 121 | 4,812,487 | $bla_{\text{ACT-25}}$, *fosA* | *gyrA* (S83I), $bla_{\text{TEM-1}}$, *ant(3'')-Ia*, *aph(3')-Ia*, *aph(3'')-Ib*, *aph(6)-Id*, *catA1*, *sul2*, *dfrA1*, *sat2* | 8 | **pCUVET19-891.1** (IncFIB: 117,320 bp): –<br>**pCUVET19-891.2** (IncFII: 69,933 bp): $bla_{\text{TEM-1}}$, *rmtB1*<br>**pCUVET19-891.3** (IncX3: 51,479 bp): $bla_{\text{OXA-181}}$, *qnrS1*<br>**pCUVET19-891.4** (no Inc: 51,004 bp): –<br>**pCUVET19-891.5** (no Inc: 3,338 bp): – |

| Strain | Species and subspecies | Host | Clinical Manifestation | Sequence type | Chromosome size | Intrinsic antimicrobial resistance genes on chromosome | Acquired antimicrobial mechanism(s) on chromosome | Number of plasmids | Plasmids (Incompatibility complex; size): Antimicrobial resistance genes |
|---|---|---|---|---|---|---|---|---|---|
| | | | | | | | | | **pCUVET19-891.6** (ColRNAI/Col440II: 3,223 bp): – **pCUVET19-891.7** (ColRNAI: 2,758 bp): – **pCUVET19-891.8** (ColRNAI: 2,495 bp): – |
| CUVET 21-1190 | *Enterobacter hormaechei* subsp. *xiangfangensis* | Cat | Peritoneal effusion | 171 | 4,678,434 | *bla*$_{ACT-45}$, *fosA* | *gyrA* (S83I), *bla*$_{CTX-M-15}$, *bla*$_{OXA-1}$, *aac(3)-IIe*, *aac(6')-Ib-cr5*, *qnrB1*, *tet*(A), *catB*, *dfrA14* | 2 | **pCUVET21-1190.1** (IncX3: 51,479 bp): *bla*$_{OXA-181}$, *qnrS1* **pCUVET21-1190.2** (ColRNAI: 2,496 bp): – |
| CUVET 21-1726 | *Enterobacter hormaechei* subsp. *xiangfangensis* | Cat | Bloodstream infection | 171 | 4,680,798 | *bla*$_{ACT-45}$, *fosA* | *gyrA* (S83I), *bla*$_{CTX-M-15}$, *bla*$_{OXA-1}$, *aac(3)-IIe*, *aac(6')-Ib-cr5*, *qnrB1*, *tet*(A), *catB*, *dfrA14* | 2 | **pCUVET21-1726.1** (IncX3: 51,479 bp): *bla*$_{OXA-181}$, *qnrS1* **pCUVET21-1726.2** (ColRNAI: 2,496 bp): – |
| CUVET 22-793 | *Enterobacter hormaechei* subsp. *steigerwaltii* | Cat | Peritoneal effusion | 65 | 4,843,472 | *bla*$_{ACT-17}$, *fosA* | – | 3 | **pCUVET22-793.1** (IncHI2/2A: 283,562 bp): *bla*$_{SHV-12}$, *bla*$_{TEM-1}$, *aac(3)-IIg*, *aac(6')-Ib3*, *aac(6')-IIc*, *aph(3'')-Ib*, *aph(6)-Id*, *arr*, *ere*(A), *catA2*, *sul1*, *dfrA19*, *mcr-9.1* **pCUVET22-793.2** (IncX3: 51,479 bp): *bla*$_{OXA-181}$, *qnrS1* **pCUVET22-793.3** (ColRNAI: 2,495 bp): – |

| Strain | Species and subspecies | Host | Clinical Manifestation | Sequence type | Chromosome size | Intrinsic antimicrobial resistance genes on chromosome | Acquired antimicrobial mechanism(s) on chromosome | Number of plasmids | Plasmids (Incompatibility complex; size): Antimicrobial resistance genes |
|---|---|---|---|---|---|---|---|---|---|
| CUVET 22-969 | *Enterobacter hormaechei* subsp. *xiangfangensis* | Dog | Bloodstream infection | 171 | 4,626,390 | *bla*$_{ACT-45}$, *fosA* | *gyrA* (S83I) | 4 | **pCUVET22-969.1** (IncX3: 54,232 bp): *bla*$_{OXA-181}$, *qnrS1* **pCUVET22-969.2** (ColRNAI/Col440II: 5,413 bp): – **pCUVET22-969.3** (ColRNAI/Col440II: 4,760 bp): – **pCUVET22-969.4** (Col440I: 2,511 bp): – |
| CUVET 18-1371 | *Serratia nevei* | Dog | Wound infection | NA | 5,412,560 | *bla*$_{SRT}$, *fosA*, *tet*(41) | *bla*$_{CMY-2}$, *aac(6′)-Ia*, *aac(6′)-Ic*, *ant(2″)-Ia* | 5 | **pCUVET18-1371.1** (IncA/C2: 109,998 bp): *bla*$_{CMY-2}$, *aac(6′)-Ia*, *ant(2″)-Ia*, *aph(3″)-Ib*, *aph(6)-Id*, *tet*(A), *floR*, *sul2* **pCUVET18-1371.2** (no Inc: 99,795 bp): – **pCUVET18-1371.3** (IncFII: 84,227 bp): *ant(3″)-Ia*, *qnrS1*, *tet*(A), *lnu*(F), *sul1*, *dfrA1* **pCUVET18-1371.4** (IncX3: 52,830 bp): *bla*$_{OXA-181}$, *qnrS1* **pCUVET18-1371.5** (ColRNAI/Col440II: 4,947 bp): – |

| Strain | Species and subspecies | Host | Clinical Manifestation | Sequence type | Chromosome size | Intrinsic antimicrobial resistance genes on chromosome | Acquired antimicrobial mechanism(s) on chromosome | Number of plasmids | Plasmids (Incompatibility complex; size): Antimicrobial resistance genes |
|---|---|---|---|---|---|---|---|---|---|
| CUVET 18-1784 | *Serratia nevei* | Dog | Cystitis | NA | 5,457,127 | *bla*$_{\mathrm{SRT}}$, *fosA*, *tet*(41) | *aac(6′)-Ic* | 5 | **pCUVET18-1784.1** (IncA/C2: 109,998 bp): *bla*$_{\mathrm{CMY-2}}$, *aac(6′)-Ia*, *ant(2″)-Ia*, *aph(3″)-Ib*, *aph(6)-Id*, *tet*(A), *floR*, *sul2*<br>**pCUVET18-1784.2** (no Inc: 99,795 bp): –<br>**pCUVET18-1784.3** (IncFII: 84,227 bp): *ant(3″)-Ia*, *qnrS1*, *tet*(A), *lnu*(F), *sul1*, *dfrA1*<br>**pCUVET18-1784.4** (IncX3: 52,830 bp): *bla*$_{\mathrm{OXA-181}}$, *qnrS1*<br>**pCUVET18-1784.5** (ColRNAI/Col440II: 4,947 bp): – |

**Table S2** Antimicrobial resistance pattern of the six OXA-181-producing *Enterobacter hormaechei* and two OXA-181-producing *Serratia nevei* isolated from dogs and cats

| Species | Strain | Antimicrobial resistance pattern* | | | | | | | | | | | | | |
|---|---|---|---|---|---|---|---|---|---|---|---|---|---|---|---|
| | | AMP | CTX | CAZ | MEM | NAL | CIP | GEN | TET | AZM | CHL | SMX | TMP | CST | TGC |
| *Enterobacter hormaechei* | CUVET18-121 | R | R | R | R | R | R | R | R | R | R | R | R | S | S |
| | CUVET19-891 | R | R | R | R | R | R | R | S | R | R | R | R | S | S |
| | CUVET21-1190 | R | R | R | R | R | R | R | R | R | I | S | R | S | S |
| | CUVET21-1726 | R | R | R | R | R | R | R | R | R | I | S | R | S | S |
| | CUVET22-793 | R | R | R | R | R | R | R | I | R | R | R | R | S | S |
| | CUVET22-969 | R | S | S | R | R | R | S | S | R | S | S | S | S | S |
| *Serratia nevei* | CUVET18-1371 | R | R | R | R | R | R | R | R | R | R | R | R | R | R |
| | CUVET18-1784 | R | R | R | R | R | R | R | R | R | R | R | R | R | R |

Note: AMP, ampicillin; AZM, azithromycin; CAZ, ceftazidime; CHL, chloramphenicol; CIP, ciprofloxacin; CST, colistin; CTX, cefotaxime; GEN, gentamicin; MEM, meropenem; NAL, nalidixic acid; SMX, sulfamethoxazole; TET, tetracycline; TGC, tigecycline; TMP, trimethoprim. Resistance was interpreted following the Clinical and Laboratory Institute (CLSI) interpretive criteria as follows: ampicillin (≥32 µg/mL), azithromycin (≥32 µg/mL), cefotaxime (≥4 µg/mL), ceftazidime (≥16 µg/mL), chloramphenicol (≥32 µg/mL), ciprofloxacin (≥1 µg/mL), colistin (≥4 µg/mL), gentamicin (≥8 µg/mL), meropenem (≥4 µg/mL), nalidixic acid (≥32 µg/mL), sulfamethoxazole (≥512 µg/mL), tetracycline (≥16 µg/mL), and trimethoprim (≥16 µg/mL) (1, 2), except for tigecycline (>0.5 mg/L) that was referred to the criteria of *E. coli* from the European Committee on Antimicrobial Susceptibility Testing (EUCAST) (3).

*Green, susceptible or S; Orange; intermediate resistance or I; Red, resistance or R.

**Table S3** Genome assembly quality of the six OXA-181-producing *Enterobacter hormaechei* and two OXA-181-producing *Serratia nevei* isolated from dogs and cats

| Strain | Species and subspecies | Genome size (bp) | GC (%) | Number of contigs | N50 (bp) | Coverage | GenBank accession number |
|---|---|---|---|---|---|---|---|
| CUVET18-121 | *Enterobacter hormaechei* subsp. *xiangfangensis* | 5,038,508 | 55.14 | 6 | 4,731,337 | 100X | CP114979-CP114984 |
| CUVET19-891 | *Enterobacter hormaechei* subsp. *xiangfangensis* | 5,114,037 | 54.98 | 9 | 4,812,487 | 100X | CP114985-CP114993 |
| CUVET21-1190 | *Enterobacter hormaechei* subsp. *xiangfangensis* | 4,732,409 | 55.17 | 3 | 4,678,434 | 100X | CP114994-CP114996 |
| CUVET21-1726 | *Enterobacter hormaechei* subsp. *xiangfangensis* | 4,734,773 | 55.17 | 3 | 4,680,798 | 100X | CP114997-CP114999 |
| CUVET22-793 | *Enterobacter hormaechei* subsp. *steigerwaltii* | 5,181,008 | 54.90 | 4 | 4,843,472 | 100X | CP115000-CP115003 |
| CUVET22-969 | *Enterobacter hormaechei* subsp. *xiangfangensis* | 4,693,306 | 55.18 | 5 | 4,626,390 | 100X | CP115004-CP115008 |
| CUVET18-1371 | *Serratia nevei* | 5,764,357 | 58.48 | 6 | 5,412,560 | 100X | CP115009-CP115014 |
| CUVET18-1784 | *Serratia nevei* | 5,808,924 | 58.53 | 6 | 5,457,127 | 100X | CP115015-CP115020 |

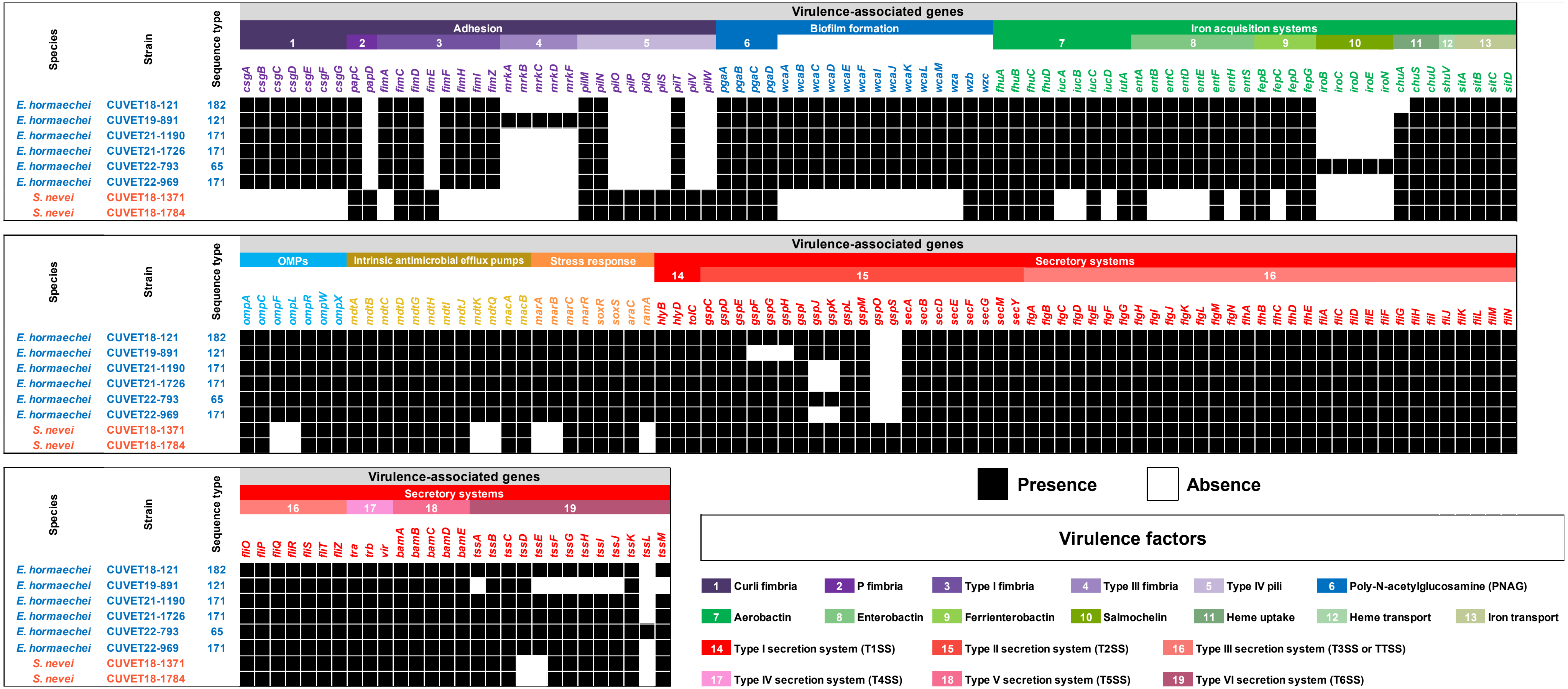


**Fig. S1** Virulence-associated genes of the six OXA-181-producing *Enterobacter hormaechei* and two OXA-181-producing *Serratia nevei* isolated from dogs and cats. The presence and absence of each gene associated with virulence factors are shown in black and white boxes, respectively.

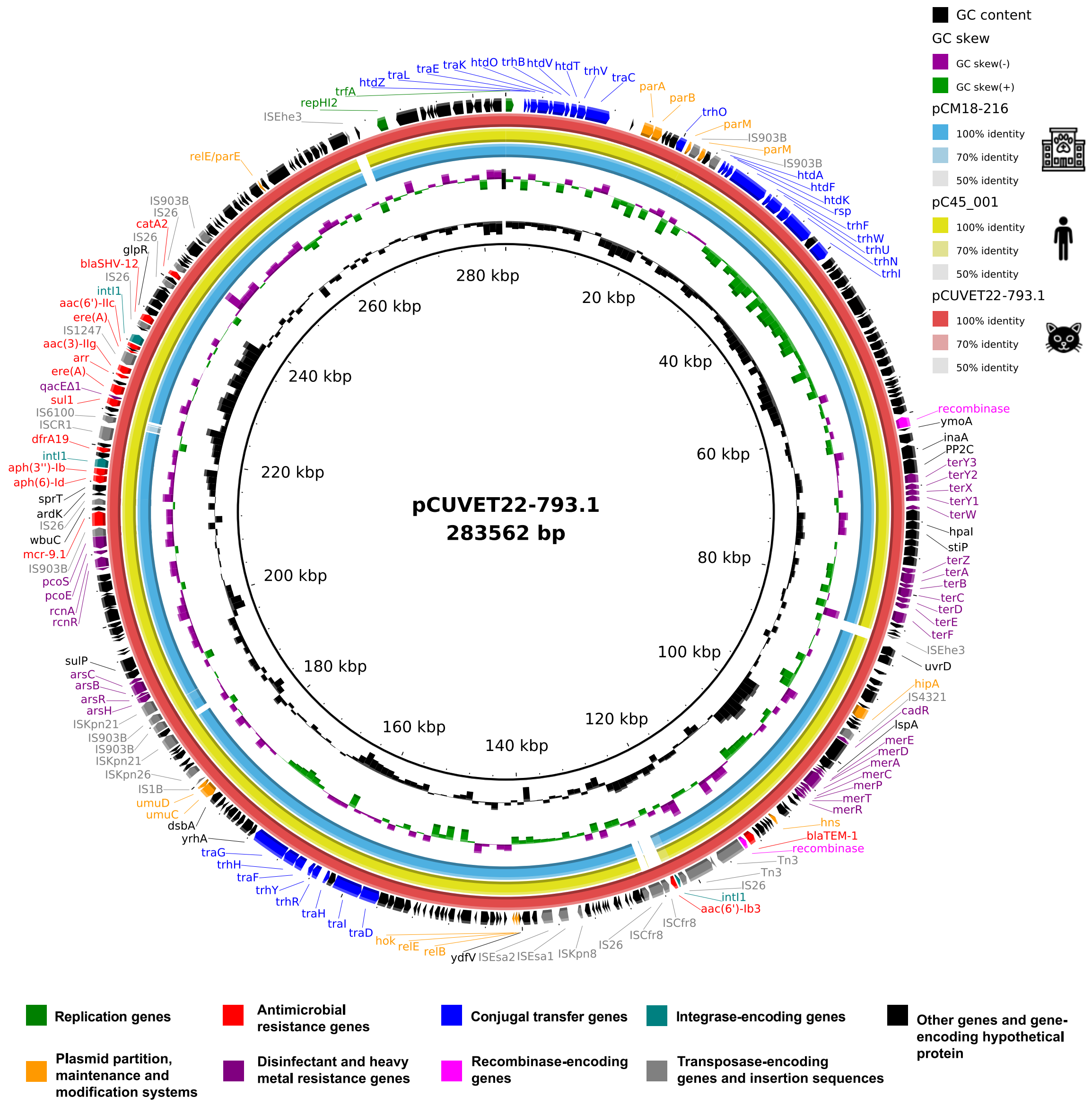


**Fig. S2** Comparison of circular map of IncHI2/2A plasmids. The two innermost rings show GC content (black) and GC skew (purple and green). The blue and yellow circles represent the IncHI2/2A plasmid in *E. hormaechei* isolated from the veterinary hospital environment (pCM18-216, GenBank accession number CP050312) and a human patient (pC45_001, GenBank accession number CP042552) in Australia, respectively. The red circle represents the same plasmid type identified in *E. hormaechei* recovered from a feline patient in this study (pCUVET22-793.1). The outermost ring illustrates the location and orientation of genes in pCUVET22-793.1. The color of each arrow represents the gene type or function as indicated in the legend at the bottom.

## REFERENCES


1. Clinical and Laboratory Standards Institute. 2023. Performance standards for antimicrobial disk and dilution susceptibility tests for bacteria isolated from animals. 6th ed. CLSI document VET01S. Clinical and Laboratory Standards Institute, Wayne, PA.
2. Clinical and Laboratory Standards Institute. 2023. Performance standards for antimicrobial susceptibility testing, 33rd ed. CLSI document M100. Clinical and Laboratory Standards Institute, Wayne, PA.
3. The European Committee on Antimicrobial Susceptibility Testing. 2023. Breakpoint tables for interpretation of MICs and zone diameters, V13.0. http://www.eucast.org.
